# Spontaneous coronary artery dissection in a patient with recent COVID‐19 infection: A case report

**DOI:** 10.1002/ccr3.6399

**Published:** 2022-10-03

**Authors:** Mohammad Javad Alemzadeh‐Ansari, Amir Akbar Fakhrabadi, Ahmad Amin, Farnaz Rafiee, Golnaz Houshmand

**Affiliations:** ^1^ Rajaie Cardiovascular Medical and Research Center Iran University of Medical Sciences Tehran Iran; ^2^ Cardiovascular Intervention Research Center, Rajaie Cardiovascular Medical and Research Center Iran University of Medical Sciences Tehran Iran

**Keywords:** ACS, case report, COVID‐19, myocardial injury, SCAD, spontaneous coronary artery dissection

## Abstract

We report a spontaneous coronary artery dissection (SCAD) case in a lady with a history of recent COVID‐19 and without any known predisposing factors. We also highlight the value of CMR as a noninvasive tool for tissue characterization, which can also be more applicable during the COVID‐19 pandemic.

## Graphical abstract

Inflammatory changes of the coronary walls in COVID‐19 may play a role in susceptibility to spontaneous coronary artery dissection. Cardiac magnetic resonance is beneficial during and after the COVID‐19 epidemic for differentiating myocardial injury.

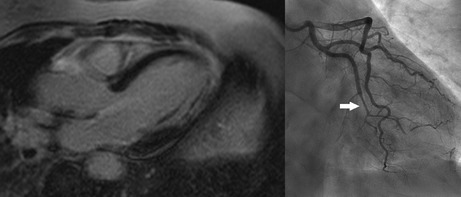

## 1 INTRODUCTION

A wide range of cardiovascular complications could affect patients with severe acute respiratory coronavirus 2 (SAR‐CoV‐2) and lead to a higher mortality rate.^1^, ^2^, ^3^ Herein, we report a spontaneous coronary artery dissection (SCAD) case in a lady with a history of recent COVID‐19.

## 2 CASE PRESENTATION

A 58‐year‐old lady who recently recovered from COVID‐19 presented with intermittent chest pain that radiated to trapezius and intra‐scapular regions during the past 2 days. The chest pain was diminished in sitting but worsened in lying back. In her active phase 2 months before, she presented with fever, gastrointestinal symptoms, and isolated severe thrombocytopenia (10‐50 × 10^3^/mm^3^), the lung involvement was minimal, and she had no respiratory symptoms. The nasopharyngeal swab for polymerase chain reaction (PCR) for SARS‐CoV‐2 was positive. She had been discharged in good condition and had a normal level platelet count.^4^ On her current admission, her blood pressure was 107/73 mmHg, heart rate 81 bpm, respiratory rate 16 breaths/min, and temperature 37.2°C. The physical examination was normal. She had a history of one pregnancy 20 years ago. She was not on hormone replacement therapy. She had not mentioned any physical or emotional stress before the event. She had hyperlipidemia but no other cardiovascular risk factors and no history of hypothyroidism.

**FIGURE 1:**
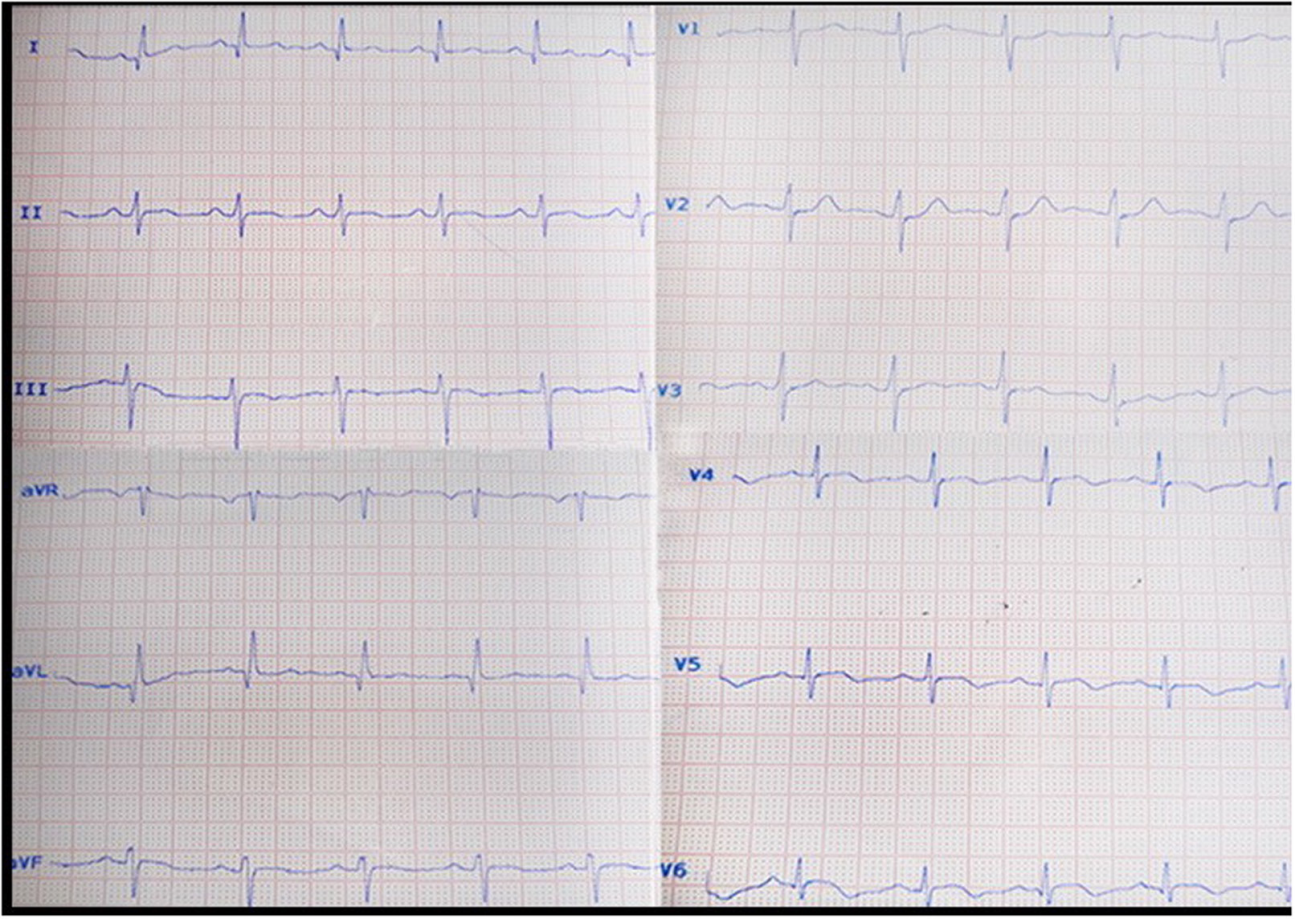
Electrocardiogram shows left anterior hemiblock with Q waves in I and aVL and invert T in V5 and V6.

**FIGURE 2:**
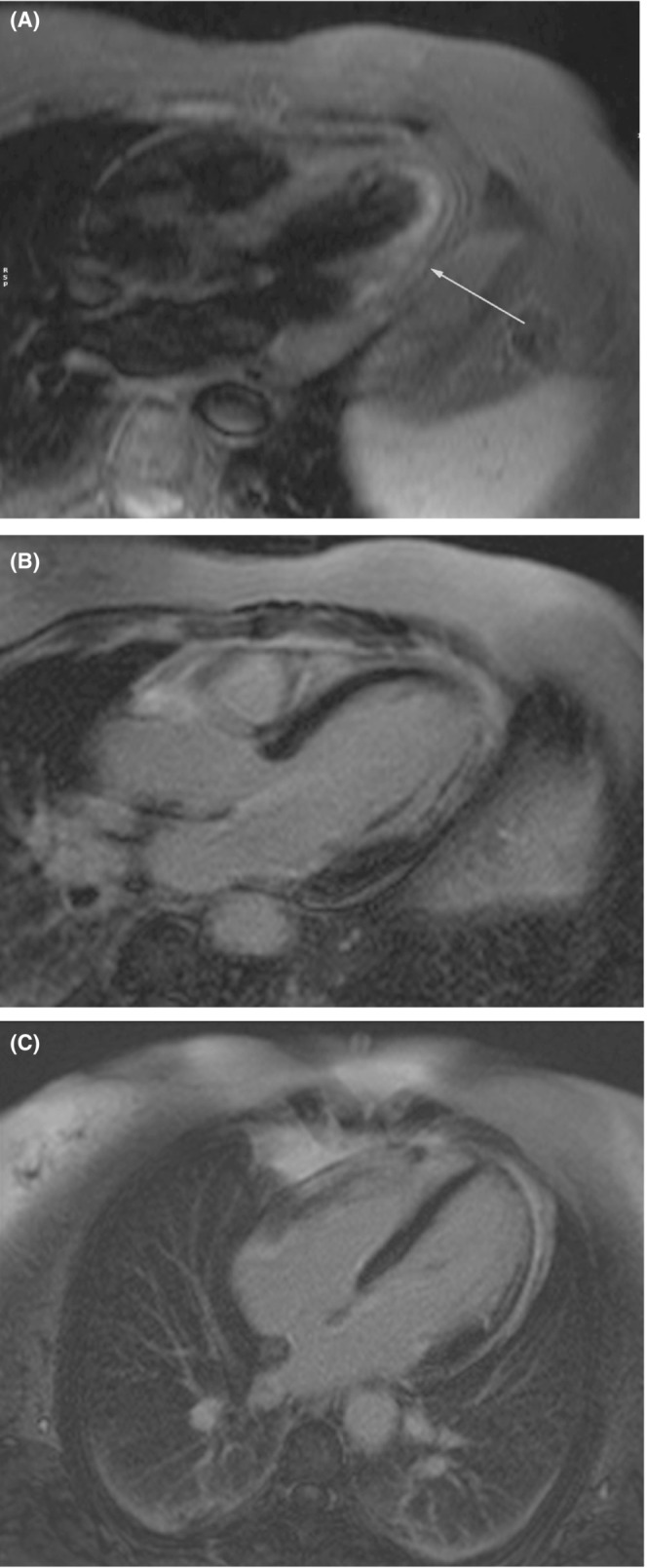
(A) T2‐weighted fat suppression image shows high intensity signal compatible with subendocardial inflammation with concomitant linear hypointensity within the area in the mid to apical lateral compatible with possible microvascular obstruction/intramyocardial hematoma (arrow). (B, C) T1‐weighted late gadolinium enhancement images show nearly transmural infarction in the mid‐lateral wall with a hypointense core in an enhanced infarct core which is concomitant microvascular obstruction/intramyocardial hematoma. There is also pericardial enhancement evident.

The 12‐lead Electrocardiography (ECG) showed Q waves in I and aVL and inverted T in V5 and V6 (Figure 1). Blood tests showed Leucocytosis white blood cell 14 × 10^9^/L with 9% lymphocyte and low normal platelet count (150 × 10^9^/L). The C reactive protein (CRP) level was high at 24 mg/dl, and there was an increased high sensitive cardiac troponin I (hs‐cTnI)19 μg/L (Normal range <0.03). Echocardiography showed mildly reduced LV function, mid‐lateral wall hypokinesia, and mild pericardial effusion. Regarding the pleuritic chest pain and the history of COVID‐19, cardiac magnetic resonance (CMR) was performed on admission day, which revealed a mildly reduced ejection fraction of 50% and akinesia in the mid to apical lateral wall. In T2‐weighted fat suppression images of short tau inversion recovery sequences (STIR), there was evidence of subendocardial inflammation with linear hypointensity area in the mid to apical lateral (Figure 2A). The late gadolinium enhancement images showed nearly transmural infarction in the mid to apical lateral with evidence of microvascular obstruction (MVO)/ Intramyocardial hematoma (IMH) hypointense core in an enhanced infarct core in line with STIR images and compatible with ischemic injury (Figure 2B,C). There is also pericardium enhancement adjacent to the lateral wall suggestive of inflammation (Figure 2B).

**FIGURE 3:**
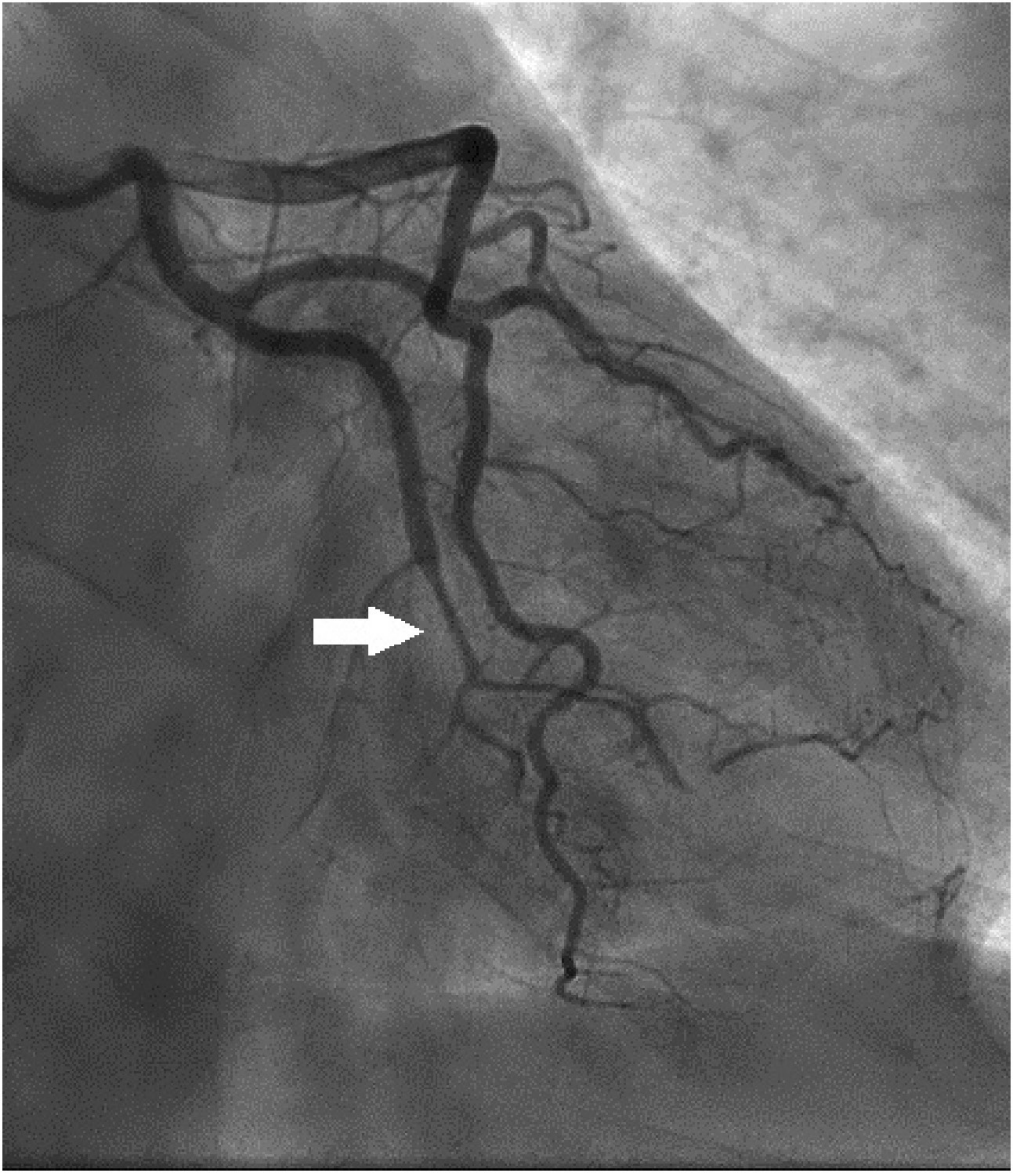
Coronary angiography shows diffuse narrowing from the mid part of LCX to the distal of terminal OM.

Coronary angiography was performed, which revealed long diffuse narrowing from the middle‐left circumflex artery (LCX) continued to the end tip of the obtuse marginal vessel (Figure 3). This is compatible with type IIB of spontaneous coronary artery dissection. The arterial flow grade was thrombolysis in myocardial infarction (TIMI) 1–2 in the affected region. The left anterior descending and right coronary arteries were normal.

We planned for medical treatment and closed follow‐up according to the size and location of the dissection and residual flow. Antiplatelet therapy with aspirin 80 mg/day, clopidogrel 75 mg/day, metoprolol 50 mg daily, lisinopril 5 mg/day, and statin 20 mg/day were prescribed as recommended by expert consensus.^5^ Polymerase chain reaction (PCR) for SARS‐COV‐2 returned negative, but the IgG was positive. Whole‐body CT angiography showed no evidence of fibromuscular dysplasia or aneurysm. Anti‐nuclear antibodies and rheumatoid factor were negative.

The patient had no specific symptoms in the three‐month and six‐month follow‐up visits and performed well under medical therapy. The inflammatory markers were within a normal limit during the 3 months follow‐up visit, and the ECG showed no further changes.

## 3 DISCUSSION

Spontaneous coronary artery dissection accounts for 4% of acute coronary syndromes and more frequently occurs in middle‐aged females. Emotional and physical stress are commonly referred to as the triggers of SCAD. Sex and hormonal changes may be influential factors. Fibromuscular dysplasia is the most common associated condition, while inherited connective tissue disorders are rare factors associated. There are two pathophysiology hypotheses proposed for coronary dissection; the first mechanism is an intimal tear and then the formation of intramural hematoma, and the second one, which is assumed to be more valid, is primary intramural hematoma due to the dissection of vasa vasorum with or without secondary intima tear.^6^ Pathologies from post‐mortem studies show eosinophilic infiltration in the adventitia of the dissected coronary vessel.^7^, ^8^ However, it remained unclear whether the inflammatory cells detected in the coronary vessel wall of a dissected coronary are primary to the injury or its consequence.^6^ Systemic inflammatory diseases, such as lupus erythematosus, and inflammatory bowel syndrome, are also suggested as a predisposing factor for coronary dissection.^6^, ^9^ However, the role of inflammation is unclear in this entity. Viral infection may cause acute coronary syndrome by triggering inflammatory processes, altered shear stress, and endothelial dysfunction.^10^ The data are limited regarding coronary dissection in the active and recovery phase of COVID‐19.^11^, ^12^, ^13^ Our patient was a middle‐aged woman, commonly seen in a setting of coronary dissection, but she had no predisposing factor that is frequently seen in these cases. The rheumatologic workup from clinical to laboratory findings was negative for any underlying collagen vascular disease. Although we cannot postulate any association due to a lack of pathologic assessment, it is hypothesized that inflammatory changes in the coronary adventitia in the setting of COVID‐19 may increase the susceptibility to coronary dissection.^14^ The ESC/ACCA position document on spontaneous coronary artery dissection proposed adventitial inflammation as one causal event of SCAD that promotes vasa vasorum micro‐vessel disruption and bleeding into tunica media.^15^ Notwithstanding, the association of recent SARS‐CoV2 infection, delayed SCAD, and any possible attributable factors require further assessment.

Our patient came with troponin‐positive chest pain with pericardial components during the pandemic. Although there was no fever, acute viral infection, or underlying inflammatory collagen vascular disease considering the recent COVID‐19 and pleuritic chest pain, myocarditis was in the differentials. Thus, we employed CMR before angiography in our diagnostic approach regarding the CMR capabilities to differentiate ischemic myocardial injury from non‐ischemic myocardial injury^16^ and lower the risk of unnecessary exposure in the cath laboratory during the COVID‐19 pandemic.

Coronary angiography is the principal modality for diagnosing coronary dissection.^6^ Intracoronary imaging has become helpful in diagnosing SCAD in circumstances where the angiogram is nondiagnostic or uncertain.^6^ However, it may impose a risk of increasing dissection length with an imaging catheter or wire.^6^, ^17^ The angiographic pattern was diagnostic for the dissection in our patient, so we did not employ intracoronary imaging not to impose any further risk of increasing dissection and ischemia considering the long dissection length and the narrow diseased vessel.

Generally, coronary interventions are associated with a high risk of complications, and according to the expert consensus, medical management is preferred over stenting in SCAD.^5^ We did not perform coronary intervention as the patient was stable, the lesion was long, and the dissection went along the distal of the vessel; the outflow at the distal would not be optimal in case of stenting, and it may lead to in‐stent thrombosis.

There is scarce evidence for optimal SCAD treatment, so the medical treatment is usually based on expert consensus. The use of single or dual antiplatelet therapy is generally suggested; beta‐blockers are commonly prescribed to relieve arterial wall stress. Patients with poor LV function or underlying FMD should consider angiotensin‐converting enzyme medications, and those with dyslipidemia should take statins.^5^ Our patient had a favorable outcome on medical treatment.

## 4 CONCLUSION

In this case report, we presented a case of SCAD in a middle‐aged female with SARS‐CoV2 history with no predisposing factor. It is hypothesized that inflammatory changes in COVID‐19 may play a role in susceptibility to SCAD. However, any association between COVID‐19 and delayed SCAD requires further assessment. Moreover, we highlight the value of CMR as a noninvasive tool for tissue characterization, which can also be more applicable during the COVID‐19 pandemic and post‐pandemic with the preference for an initial noninvasive approach to differentiate myocardial injury.

## AUTHOR CONTRIBUTIONS

MJAA and AAF contributed to the writing of the manuscript. AA and FR contributed to collecting the data and writing the manuscript. GH contributed to the writing of the manuscript and critical reviewing. All authors have read and approved the manuscript.

## FUNDING INFORMATION

No funding was obtained for this study.

## CONFLICT OF INTEREST

The authors declare that there is no conflict of interest.

## ETHICAL APPROVAL

Not applicable.

## CONSENT

Written informed consent was obtained from the patient to publish this case report and any accompanying images.

## Data Availability

All data supporting the conclusions are presented in the manuscript.
